# Supplementary material for: Electrical resistance of individual defects at a topological insulator surface
Source: Nat Commun. 2017 Jun 12;8:15704. doi: 10.1038/ncomms15704 (PMC5472778; doi:10.1038/ncomms15704)
Supplement: Supplementary Information — Supplementary Figures, Supplementary Notes and Supplementary References [file ncomms15704-s1.pdf]

Supplementary Note 1: Angle resolved photoemission spectroscopy

A cut in the $k_{\parallel,x}/k_{\parallel,y}$ plane at the Fermi energy and $h\nu = 8.4$ eV is shown in Supplementary Fig. 1 (a). We observe a small amount of warping of the Dirac cone and an average Fermi wave vector of $k_\mathrm{F} = 0.07(1) \cdot 10^{10}\ \mathrm{m}^{-1}$ which we determined by fitting a circle to the Fermi surface. Note: The asymmetry of the Fermi surface stems from the geometrical arrangement of the ARPES measurement setup. Hereby, the k_y direction is scanned by rotation of the sample with respect to the photon source. Due to the analyzer sitting in a fixed 45° angle with respect to the photon source, the resulting intensity in $-\mathrm{k}_y$ direction is higher than in $+\mathrm{k}_y$ direction. The corresponding dispersion in $k_{\parallel,x}$ is shown in Supplementary Fig. 1 (b). Supplementary Fig. 1 (c) shows spin-resolved energy distribution curves measured with a FeO-based spin detector (FERRUM, Focus GmbH) along the yellow box indicated in Supplementary Fig. 1 (b). The resulting in-plane spin polarization shown in Supplementary Fig. 1 (d) is as high as 50% in the Dirac cone indicating the topological nature of the surface state. Supplementary Fig. 1 (e) shows a cut in the $k_{\parallel,x}/k_{\parallel,y}$ plane at the Fermi energy measured at $h\nu = 21.2$ eV. At this photon energy, the relative intensity of the TSS is smaller while that of the bulk bands is higher in comparison to measurements at $h\nu = 8.4$ eV[1]. Here, we observe a small contribution from the bulk conduction band at the Fermi energy from which we conclude that the Fermi energy lies just at the conduction band edge. The corresponding dispersion in $k_{\parallel,x}$ is shown in Supplementary Fig. 1 (f) where the TSS is also not visible due to its low photoemission cross section. From this measurement, we extract the band gap to be $260(20)$ meV. The carrier concentration in the TSS can be determined from k_F after Supplementary Ref. [2]

$$n_\mathrm{surf}(E_\mathrm{F}) = \frac{k_\mathrm{F}^2}{4\pi} = \frac{(0.07(1) \cdot 10^{10}\ \mathrm{m}^{-1}\ )^2}{4\pi} = 4(1) \cdot 10^{12}\ \mathrm{cm}^{-2}, \qquad (1)$$

where k_F is taken from the $k_{\parallel,x}/k_{\parallel,y}$ plane measurement in Supplementary Fig. 1 (a).

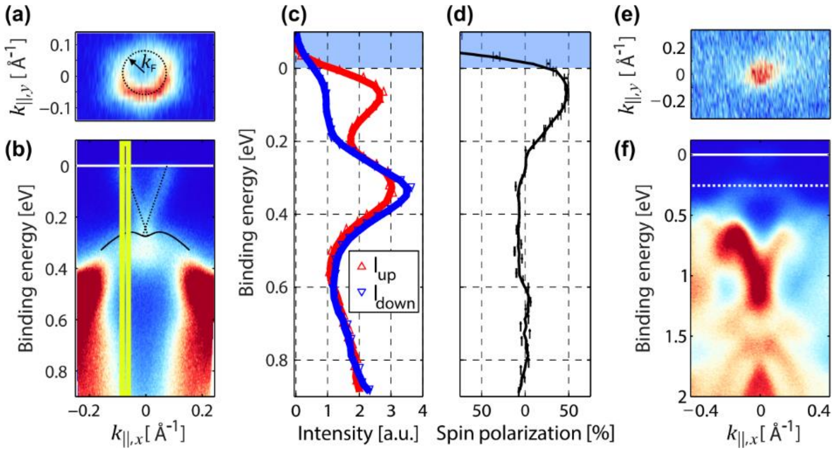

Supplementary Figure 1. Angle resolved photoemission spectroscopy. (a) Cut in the $k_{\parallel,x}/k_{\parallel,y}$ plane at the Fermi Energy ($h\nu = 8.4$ eV). (b) Corresponding dispersion in $k_{\parallel,x}$ direction with the Dirac cone indicated by dotted lines. (c) Spin-resolved energy distribution along the yellow box in (b). (d) Relative spin polarization resulting from (c). (e) Cut in the $k_{\parallel,x}/k_{\parallel,y}$ plane at the Fermi energy ($h\nu = 21.2$ eV). (f) Corresponding dispersion in $k_{\parallel,x}$ direction with valence band edge indicated by the dotted line.

Supplementary Note 2: Potentiometry voltage feedback

Shown in Supplementary Fig. 2 is the I/V curve of the tunneling tip around $V_{\text{tip}} = 0$ V where we find an almost linear dependence with finite slope. In this example, the stabilization parameters for the topography feedback were $V_{\text{tip}} = -10$ mV and $I_T = 100$ pA. During the potentiometry feedback, the tip is held at constant height above the sample surface and the tip voltage is adjusted, resulting in a change of the tunneling current corresponding to the shown I/V curve. The tip voltage is adjusted by the feedback loop until a current $I_T = 0$ A is reached such that the tip

potential is the same as the underlying sample. More details on the method can be found in Supplementary Ref. [3].

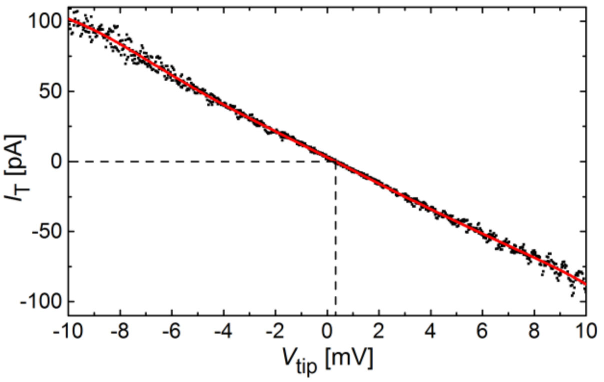

Supplementary Figure 2. Current voltage characteristics of the tunneling tip. The I/V curve around $V_{\mathrm{tip}} = 0$ V shows an almost linear slope across the voltage range. At $I_{\mathrm{T}} = 0$ A a finite voltage is observed corresponding to the local sample potential under the tip.

Supplementary Note 3: Voltage drop upon reversal of the current direction.

Supplementary Fig. 3 shows a potentiometry section for reverse current directions resulting in the reverse voltage drop. We do not observe a dependence of the absolute voltage drops at steps, domain boundaries and across terraces on the current direction within the measurement errors.

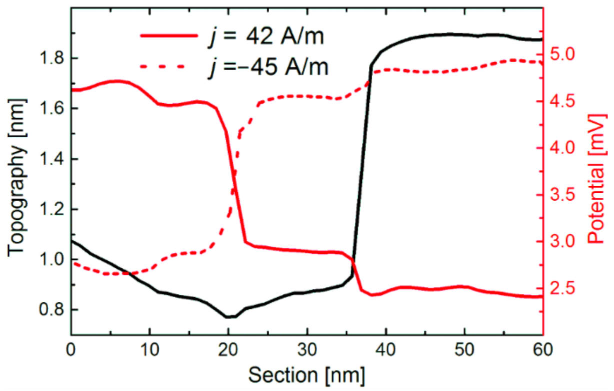

Supplementary Figure 3. Comparison of voltage drop for reverse current directions. The current densities are $j = 42(9)\ \mathrm{A\,m^{-1}}$ and $j = -45(9)\ \mathrm{A\,m^{-1}}$, respectively. Only small differences in the absolute voltage drops for both current directions are visible which we attribute to uncertainties within the measurement such as the exact position of the tips and small variations in the current density.

Supplementary Note 4: Resistor network calculations.

In order to validate the results from the resistor network calculations, we compare the two-dimensional analytic solution around a circular defect after Supplementary Ref. [4] to the results obtained from the resistor network calculations. Hereby, the resistor network should lead to the same result as the classical analytic description for a large enough system compared to the defect size and fine enough mesh of resistors.

For a small defect size compared to the system size (10 nm defect diameter compared to 1 µm system size with 200×200 nodal points) the potential distribution resulting from the resistor network is shown in Supplementary Fig. 4 (a). The corresponding analytic solution and resistor network voltage curves, shown in Supplementary Fig. 4 (b), almost coincide with only a notable difference of below 5% very close to the defect. We conclude that the analytic solution and resistor network give the same results for a large enough system and that the number of resistors in the

system is sufficient. On the other hand, Supplementary Fig. 4 (c) shows the potential distribution resulting from the resistor network for a larger defect (100 nm diameter compared to 1 µm system size again with 200 × 200 nodal points). In this case, a significant difference between the analytic solution and the resistor network curve is evident as shown in Supplementary Fig. 4 (d). Here, in the vicinity of the defect the difference of the two curves is about 40% of the dipole amplitude which is a direct result of the finite size of the resistor network which cannot be described by the analytic solution. In conclusion, large resistor networks, compared to the size of the defect, can be used to reproduce the results of the analytic solution while small resistor networks can be used to evaluate finite size effects. Furthermore, resistor networks can describe the potential distribution around arbitrarily shape defects, which makes them even more versatile concerning the analysis of experimental data.

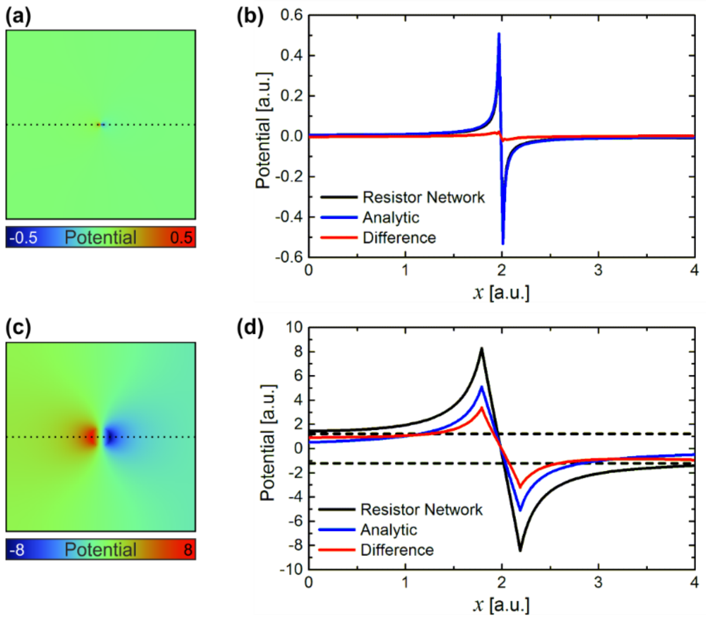

Supplementary Figure 4. Resistor network calculations compared to analytic theory. (a) Potential distribution around a small defect resulting from the resistor network (linear background subtracted). (b) Plot of the section indicated in (a), analytic potential curve and difference between

resistor network and analytic curve. (c) Potential distribution around a larger defect in a resistor network of the same size as in (a) (linear background subtracted). (d) Plot of the section indicated in (c), analytic potential curve and difference between resistor network and analytic curve.

SUPPLEMENTARY REFERENCES

[1] Plucinski, L., Herdt, A., Fahrendorf, S., Bihlmayer, G., Mussler, G., Döring, S., Kampmeier, J., Matthes, F., Bürgler, D. E., Grützmacher, D., Blügel, S., and Schneider, C. M. Electronic structure, surface morphology, and topologically protected surface states of Sb_2Te_3 thin films grown on Si(111). *J. Appl. Phys.* **113**, 053706 (2013).

[2] Zhang, J., Chang, C.-Z., Zhang, Z., Wen, J., Feng, X., Li, K., Liu, M., He, K., Wang, L., Chen, X., Xue, Q.-K., Ma, X., and Wang, Y. Band structure engineering in $(Bi_{1-x}Sb_x)_2Te_3$ ternary topological insulators. *Nat. Commun.* **2**, 574 (2011).

[3] Lüpke, F., Korte, S., Cherepanov, V., and Voigtländer, B. Scanning tunneling potentiometry implemented into a multi-tip setup by software. *Rev. Sci. Instrum.* **86**, 123701 (2015).

[4] Feenstra, R. and Briner, B. The search for residual resistivity dipoles by scanning tunneling potentiometry. *Superlattices Microstruct.* **23**, 699 – 709 (1998).
